# Profiling the role of microorganisms in quality improvement of the aged flue-cured tobacco

**DOI:** 10.1186/s12866-022-02597-9

**Published:** 2022-08-15

**Authors:** Xinying Wu, Wen Cai, Pengcheng Zhu, Zheng Peng, Tianfei Zheng, Dongliang Li, Jianghua Li, Guanyu Zhou, Guocheng Du, Juan Zhang

**Affiliations:** 1 School of Biotechnology, Jiangnan University, 1800 Lihu Road, Wuxi, 214122 China; 2 Science Center for Future Foods, Jiangnan University, 1800 Lihu Road, Wuxi, 214122 China; 3 School of Liquor and Food Engineering, Guizhou University, Guiyang, 550025 China; 4 Technical Research Center, China Tobacco Sichuan Industrial Co., Ltd., 56 Chenglong Road, 610000 Chengdu, China; 5 The Key Laboratory of Carbohydrate Chemistry and Biotechnology, Ministry of Education, Jiangnan University, 1800 Lihu Road, Wuxi, 214122 China

**Keywords:** Metabolome, Microbiome, Multi-omics integrated analysis, Quality improvement, Flue-cured tobacco, Aging process

## Abstract

**Background:**

The aging process in the tobacco production, as in other food industries, is an important process for improving the quality of raw materials. In the spontaneous aging, the complex components in flue-cured tobacco (FT) improve flavor or reduce harmful compounds through chemical reactions, microbial metabolism, and enzymatic catalysis. Some believed that tobacco-microbe played a significant part in this process. However, little information is available on how microbes mediate chemical composition to improve the quality of FT, which will lay the foundation for the time-consuming spontaneous aging to seek ways to shorten the aging cycle.

**Results:**

Comparing aged and unaged FT, volatile and non-volatile differential compounds (DCs) were multi-dimensionally analyzed with the non-targeted metabolomes based on UPLC-QTOP-MS (the ultra-performance liquid chromatography quadrupole time-of-flight mass spectrometry), GC–MS (gas chromatography-mass spectrometer) assisted derivatization and HP-SPME-GC/MS (headspace solid-phase micro-extraction assisted GC–MS). Products associated with the degradation pathways of terpenoids or higher fatty acids were one of the most important factors in improving FT quality. With the microbiome, the diversity and functions of microbial flora were analyzed. The high relative abundance function categories were in coincidence with DCs-related metabolic pathways. According to the correlation analysis, *Acinetobacter*, *Sphingomonas* and *Aspergillus* were presumed to be the important contributor, in which *Aspergillus* was associated with the highest number of degradation products of terpenoids and higher fatty acids. At last, the screened *Aspergillus nidulans* strain F4 could promote the degradation of terpenoids and higher fatty acids to enhance tobacco flavor by secreting highly active lipoxygenase and peroxidase, which verified the effect of tobacco-microbes on FT quality.

**Conclusions:**

By integrating the microbiome and metabolome, tobacco-microbe can mediate flavor-related substances to improve the quality of FT after aging, which provided a basis for identifying functional microorganisms for reforming the traditional spontaneous aging.

**Supplementary Information:**

The online version contains supplementary material available at 10.1186/s12866-022-02597-9.

## Background

Aging is an essential process for improving the quality of raw material in food industries, including fermented meat [1], wine [2], tea [3] etc. Aging treatment will produce a more intense flavor and reduce the irritation in raw material, but the spontaneous aging is a time-consuming process because of less efficiency [4]. Therefore, it is significant to understand the role of microorganisms in quality improvement and seek ways to speed up the aging process through artificial intervention.

Tobacco (*Nicotiana tabacum L.*) is one of the global cash crops and the main raw material for tobacco production. Flue-cured but unaged tobacco does not qualify as a raw material because of its irritating and undesirable flavor [5]. Then flue-cured tobacco (FT) must be aged for two years under natural conditions (20 °C-30°C, the relative humidity at 65%-75%). During aging, chemical compositions in FT develop flavor or reduce harmful compounds through chemical interactions, microbial metabolism, and enzymatic catalysis. Predecessors suggested microorganisms played an important role in this process, and have studied the microbial diversity [5, 6]. However, there is little information about the role of microorganisms in mediating the chemical composition to improve the quality of FT by aging.

With the development of non-targeted metabolomics, it is possible to obtain extensive data to profile the chemical diversity of FT. Metabolomics based on UPLC-QTOP-MS (the ultra-performance liquid chromatography quadrupole time-of-flight mass spectrometry) can identify non-volatile compounds associated with flavor precursors and based on GC–MS (gas chromatography-mass spectrometer) can identify volatile compounds related to aroma [7]. GC–MS assisted derivatization can identify compounds with strong polar or poor thermal stability. Non-targeted metabolomics based on UPLC-QTOP-MS have been used to investigate the differences in compounds in tobacco growth stages [8, 9], which provided a good reference for the chemical diversity of FT in aging. FT has a unique and complex flavor profile due to the combined action of over 4,000 compounds [9]. Consequently, the integration of metabolomes on different assay platforms will get a good metabolite coverage. In the meantime, the colonization of microflora may mediate the compositional changes, thereby altering the sensory quality of FT. With high throughout sequencing technology, Illumina MiSeq sequencing can reveal bacterial and fungal diversity of FT more comprehensively than previous studies [6, 7]. By integrating the microbiome and metabolome, correlation analysis is a dominant method for profiling the relationships between important microorganisms and flavor-related compounds. Li JJ et al. [10] applied an integration analysis to explored the optimum fermentation conditions of tobacco. Wang et al. [11] identified the flavor-producing core microbiota according to the number of flavors highly correlated in traditional Chinese vinegar. Huang et al. [12] profiled the causes of sugarcane bitterness and found that six microbial genera played a major metabolic role based on the correlation-based network analysis of microbes and metabolites.

In the present study, by comparing the aged flue-cured tobacco (AFT) to the unaged flue-cured tobacco (UAFT) samples, the differences of compounds diversity were multi-dimensionally analyzed with the non-targeted metabolomes based on three different assay platforms, the differences of microbial diversity and their metabolic functions were analyzed using advanced high-throughput sequencing technology. At last, by establishing correlation analysis between the differential compounds (DCs) and the dominant genera, the important genera were identified and their role in improving FT quality by bioaugmentation was verified.

## Results

### Profiling of DCs related to quality improvement after aging

**Fig. 1:**
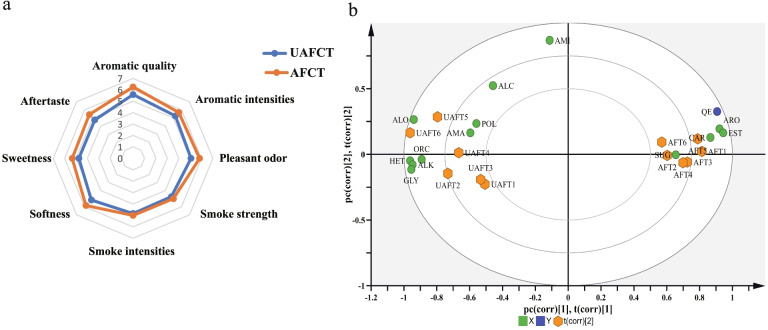
Differential analysis between the quality evaluation and DCs of UAFT and AFT samples. **a** radar map of quality traits. **b** scatter plot of the quality traits and the classification of DCs based on OPLS-DA model. ARO, aromatic compounds; ALO, alkanes and olefins; ORC, organic acid; AMA, amino acid; HET, heterocycle; POL, polyphenol; ALK, alkaloid; AMI, amine; EST, Esters and lactones; ALC, alcohol; CAR, carbonyl compounds; SUG, sugar; GLY, glycosides; QE, quality evaluation

Using the non-targeted metabolomes, we detected 983 non-volatile compounds by UPLC-QTOP-MS, 68 compounds by GC–MS assisted derivatization, and 210 volatile compounds by HP-SPME-GC/MS (headspace solid-phase micro-extraction assisted GC–MS). Total ion chromatograms were shown in Fig. A1. After two-year aging, scores of the quality traits of AFT were higher than ones of UAFT and the total evaluation score increased by 6 points (Fig. 1a). According to OPLS-DA (Orthogonal Partial Least Squares Discriminant Analysis) models (Fig. A2), the samples of UAFT and AFT were distinctly different in the horizontal direction and all samples were in a 95% confidence interval. The 200 response permutation tests showed that the OPLS-DA models did not over-fit and were valid with good prediction (Fig. A3). Based on VIP (variable importance in the projection) value > 1.0 and *p* (probability) value < 0.05, 233 DCs related to quality change were selected out, among which 19, 70 and 144 DCs came from OPLS-DA models of GC–MS assisted derivatization, HP-SPME-GC/MS and UPLC-QTOP-MS, respectively (Table A1). DCs were including 31 acids, 31 carbonyls, 30 esters and lactones, 29 heterocyclic compounds, 18 alkaloids, 14 amine, 13 amino acids, 9 saccharides, 7 glycosides and others. According to the OPLS-DA score plot (Fig. 1b), carbonyls, esters and lactones, and saccharides positively correlated with the quality evaluation, whereas the amino acids, alkaloids, and glycosides negatively correlated with the quality.

**Fig. 2:**
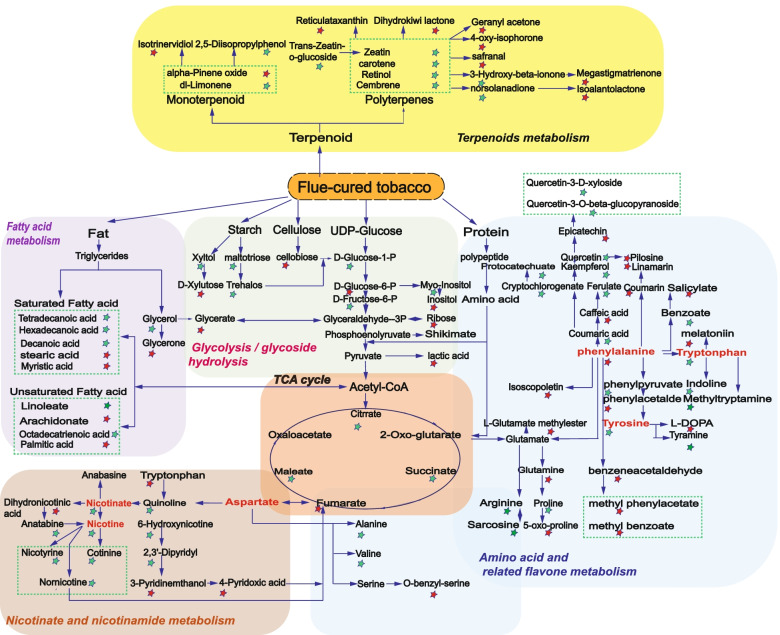
Scheme of the metabolic pathways involved in the changes of the selected DCs after aging. Italics and different color words are metabolic pathway names; Red stars represent compounds with up-regulated content; Green stars represent compounds with down-regulated content; Green dotted area is the same class of compounds

**Fig. 3:**
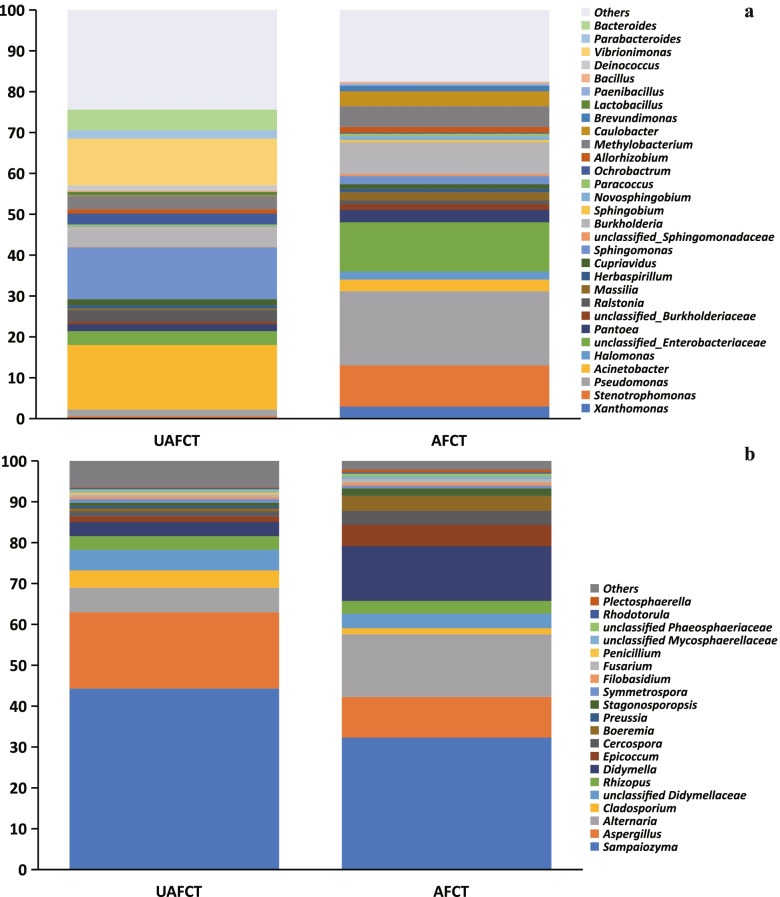
Bacterial (**a**) and fungal (**b**) diversity analysis in UAFT and AFT samples

DCs were traced to the associated metabolic pathways based on the KEGG (Kyoto Encyclopedia of Genes and Genomes) database (http://www.genome.jp/kegg/pathway.html). The differential metabolic pathways with some selected DCs were integrated and visualized in Fig. 2. Except glycolysis and the tricarboxylic acid cycle pathways, there were two types of pathways. Fatty acid metabolism, terpenoids metabolism, and amino acid/related flavone metabolism are some pathways associated with flavor formation. Nicotinate and nicotinamide metabolism associated with the degradation of harmful substances of FT. The formation of flavor was the important factor in improving the FT quality.

### Profiling of microbial diversity of FT after aging

By high-throughput sequencing, there were 825,946 V4-V5 16S rRNA and 395,875 ITS1 sequence reads generated from six samples, which were the sum of three parallel samples from both UAFT and AFT groups. The amplicon sequence variants (ASVs) are considered more precise than the operational taxonomic units (OTUs) promoted by the current mainstream analysis platform (QIIME2) (https://docs.qiime2.org/2019.7/tutorials/overview/). There was a total 601,451 ASVs for bacteria and 345,127 ASVs for fungi (Table A2 and A3). After the data of ASVs was flattened, the microbial diversity was analyzed at the same sequencing depth. The diversity of bacteria and fungi was visualized in Fig. 3. After aging, *Pseudomona*, *unclassified_Enterobacteriaceae, Stenotrophomonas* and *Alternaria, Didymella, Aspergillus* remained over 10% relative abundance. Comparing AFT to UAFT (*p* value < 0.05), *Pseudomona, unclassified_ Sphingomonadaceae, Sphingobium and unclassified Burkholderiaceae* significantly increased, whereas *Acinetobacter, Burkholderia, Vibrionimonas, Ochrobactrum, Deinococcus* significantly reduced after aging. In the fungal community, *Sampaiozyma*, *Penicillium, Aspergillus* and *Cladosporium* significantly decreased, whereas *Alternaria, Epicoccum, Plectosphaerella, Cercospora* and *Boeremia* were found to be significantly more abundant. *Unclassified_Sphingomonadaceae*, *Didymella, Epicoccum, Cercospora* and *Plectosphaerella* were extremely significant difference between the two samples (*p* < 0.001). According to the alpha diversity indexes of the flora (Table A4 and A5), Goods_coverage indexes were above 99%, meaning that the sequencing had a high coverage for each sample and all alpha diversity indices had increased after the aging process.

**Fig. 4:**
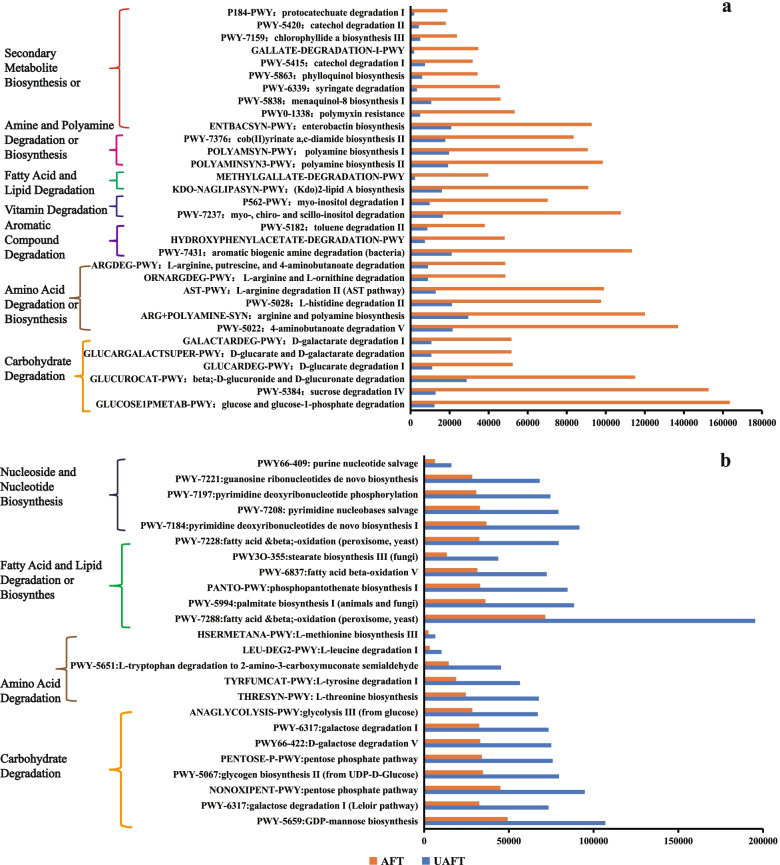
Comparison of function modules between UAFT and AFT in the level 3 MetaCyc pathway. **a** bacterial function modules; **b** fungal function modules

Metabolic functions of the microbial community were predicted based on the relative abundance of marker gene using PICRUSt2 software (Phylogenetic Investigation of Communities by Reconstruction of Unobserved States) and the MetaCyc database. After normalization of the raw data, 138,696 and 39,265 read counts per million (CPM) were obtained for functional predictions of bacterial and fungal flora, respectively. Seven and five first-level Metacyc functions and 49 and 26 s-level Metacyc functions were observed in bacterial and fungal flora, respectively (Table A6 and A7). Some third-level MetaCyc pathways with high relative abundance were shown in Fig. 4. Bacterial and fungal functional modules mainly include carbohydrate degradation, amino acid metabolism, aromatic compound metabolism, fatty acids and lipids metabolism, amine and polyamine metabolism, secondary metabolite metabolism and so on. It is easy to see that the bacterial functions were significantly enriched, whereas fungal functions showed the opposite trends after the aging process, which indicates that the metabolism of the bacteria remains quite active after two-year aging. The high relative abundance of carbohydrate degradation is fundamental to maintaining the survival of microorganisms. In the bacterial functional modules, amino acid metabolism, aromatic compound metabolism, fatty acid and lipid metabolism, secondary metabolite metabolism and so on, have a higher relative abundance than other pathways. However, the fatty acid and lipid metabolism had the highest relative abundance in the fungal modules. These dominant metabolic pathways are usually closely related to the formation of flavor or aroma. It was noteworthy that the dominant functions of microbial communities had performed in coincidence with DCs-related metabolic pathways. This meant that microbiomes mediated changes in the chemical composition of the ageing FT.

### Correlation analysis between the dominant genera and DCs

**Fig. 5:**
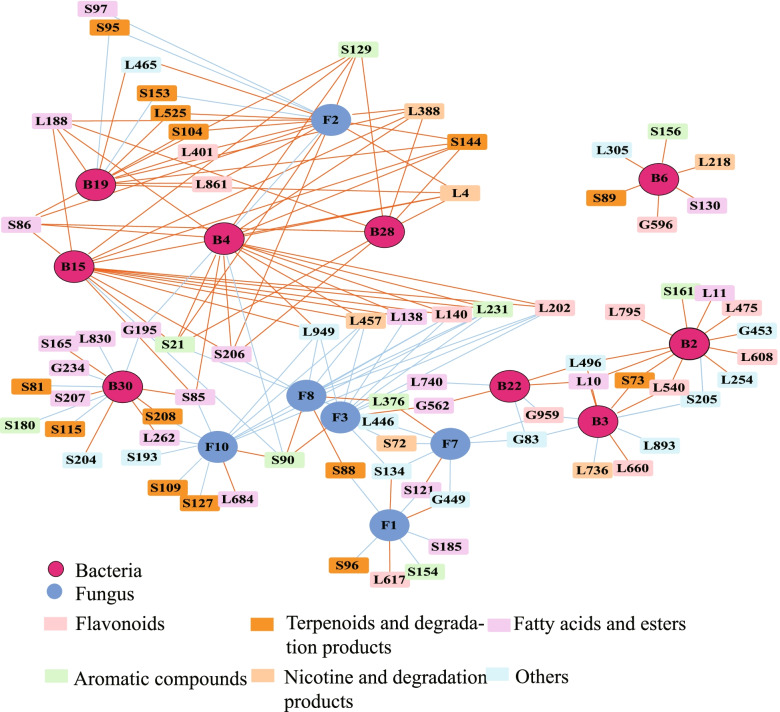
Correlation analysis between the dominant genera and significant DCs between the UAFT and AFT samples. The rounded nodes represent the genera and the square nodes represent DCs. The orange line is a positive correlation, and the blue line is a negative correlation. B2, *Stenotrophomonas*; B3, *Pseudomonas*; B4, *Acinetobacter*; B6, *unclassified_Enterobacteriaceae*; B15, *Sphingomonas*; B19, *Ochrobactrum*; B22, *Caulobacter*; B28, *Vibrionimonas*; F1, *Sampaiozyma*; F2, *Aspergillus*; F3, *Alternaria*; F7, *Didymella*; F8, *Epicoccum*; F10, *Boeremia*; B30, *Bacteroides.* **Flavonoids:** L475, proacacipetalin; L540, flazine; L608, isoscopoletin; L617, kaempferol-3-O-hexoxyl-hexoside; L660, mahaleboside; L795, dihydropanaxacol; L861, quercetin; G596, caffeic acid; G959, chlorogenic acid; L401, artemisene. **Terpenoids and degradation products:** L525, erogorgiaene; S88, dihydroactinidiolide; S95, megastigmatrienone A; S96, safranal; S109, β-Cyclocitral; S115, megastigmatrienone B; S144, linalool; S127, trans-geranylgeraniol; S153, 3-hydroxy-β-damascone; S208, limonene. **Fatty acids, esters and relatives**: L10, isostearic acid; L11, ( +)-myrtenyl formate; L188, 2E,8E-dodecadienoic acid; L262, y-linolenic acid; L684, muramic acid; L740, n-propyl carbamate; L830, picrasin; G195, glycerol; G234, glycerate; G562, palmitic acid; S85, 2-methyl-5-oxotetrahydrofuran-2-carboxylic acid; S86, dodecane; S97, propanoic acid butyl ester; S121, benzeneacetic acid methyl ester; S130, hexanoic acid methyl ester; S165, 2,8-methyl undecane; S185, 3-methyl pentadecane; S206, 4-methyl-nonane; S207, 3-methyl undecane. **Aromatic compounds:** L231, 3-amino-2-naphthoic acid; L376, alpha-methylphenylalanine; S21, 2-methyl-benzaldehyde; S90, benzeneacetaldehyd; S129, 1-phenyl-ethenone; S154, 2,4-dimethyl-benzaldehyde; S156, 1,2,3,4-tetrahydro-5,6,7,8-tetramethyl-Naphthalene; S161, 2,2',5,5'-tetramethyl-1,1'-Biphenyl; S180, α-ethylidene-benzeneacetaldehyde. **Nicotine and degradation products:** L4, nicotine; L218, 3,6-dihydronicotinic acid; L388, anatabine; L457, cotinine; L736, nornicotine; S72, 3-(1-methyl-1H-pyrrol-2-yl)- pyridine. **Others:** L134, 1-Naphthylamine; L138, glycerol phosphate choline; L140, 2-Aminoethyl indole; L202, 2-Methylindole; L254, 3'-Sialyllactose; L305, 5-oxo-L-proline; L446, choline; L465, D-asparagine; L496, dihydrolysergic acid amide; L893, sarcosine; L949, tryptophan; G83, alanine; G453, D-galactose; S134, 2-methyl-5-isopropenylfuran; S193, trans-dimethylaminocinnamonitrile; S204,2,2,4,4-tetramethyloctane; S205, 2,3-Dimethyl maleic anhydride

The differential genera, including the nine bacterial and six fungal genera, were assessed by the OPLS-DA (Fig. A4, Table A8). A correlation analysis was performed in order to profile the affinity of the relationship between the differential genera and DCs. As shown in Fig. 5, there were 161 good correlations (|*r*|> 0.7 and *p* < 0.05). DCs marked with different colors were classified according to the related metabolic pathways shown in Fig. 2, in which there are 19 fatty acids/esters and relatives, 10 flavonoids, 10 terpenoids and degradation products, 9 aromatic compounds, 6 nicotine and degradation products and others. The important genera were identified based on the number of highly correlated DCs. Notably, *Acinetobacter* (B4), *Sphingomonas* (B15) and *Aspergillus* (F2) were ranked in the top three flora because of their association with more than 16 DCs. Among three strains, strain F2 is associated with the highest number of DCs, which are mainly terpenoids and their degradation products (megastigmatrienone, 3-hydroxy-β-damascone, erogorgiaene, β-cyclic citral, linalool) and fatty acids/esters (2E,8E-dodecadienoic acid, y-linolenic acid, dodecane, propanoic acid butyl ester, glycerol, 4-methyl-nonane). Like other plants, terpenes are very important precursors of aroma and flavor in tobacco, especially carotenoids. So, the degradation of terpenoids can contribute to the quality of tobacco aroma or flavor. Fatty acids/esters also impart a pleasant odor, as in fruits. The accumulation of these substances is closely related to the enhancement of the aroma quality of FT, as described in Fig. 1 a (traits of aroma quality and pleasant odor were significantly increased after aging). Moreover, *Aspergillus* is versatile due to its ability to secrete a wide range of enzymes and widely dispersed in nature. Therefore, we screened *Aspergillus spp.* as the research object to verify the effect of microorganisms on tobacco quality.

### Validation of the role of important microorganisms

**Fig. 6:**
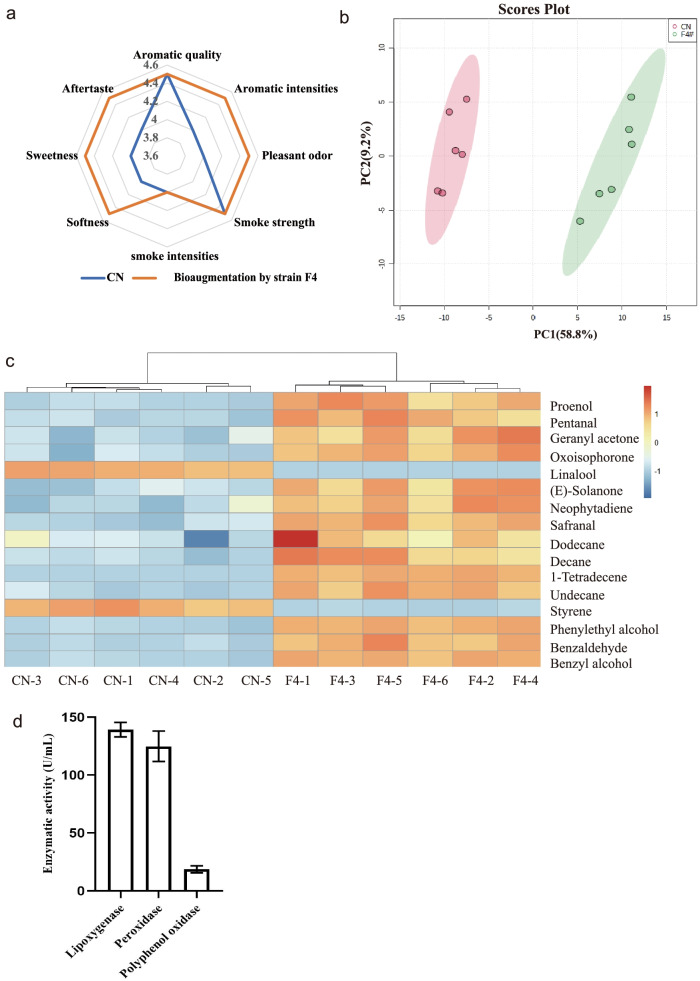
Profiling the difference between bioaugmentation by F4 and control sample (CN). **a** radar map of quality traits; **b** score plot of PCA based on the relative content of volatile components; **c** heat map of DCs-related flavor; **d** analysis of enzyme activity of strain F4

With the aid of the flow cytometric sorting techniques, we obtained the cultivable filamentous strains from the aged FCT, in which strain F4 has a high frequency of emergence. In the primary screening, the effect of the filamentous strains on the color of the medium was compared with the un-inoculated sample at 460 nm by spectrophotometry. Flavonoids, polyphenols and polyterpenoids of tobacco contribute to the color of the tobacco extract in the primary screening media. Therefore, the lighter color of the medium is related to the ability of the microorganisms to metabolize the FT extract. Then, we could easily obtain filamentous fungi that might be improve the quality of flue-cured tobacco by degrading terpenoids through the primary screening. Further, through the molecular biology identification and the bioaugmentation with strains, *Aspergillus* with the ability to improve the quality of FT can be obtained. At last, we selected out *Aspergillus nidulans* strain F4 from the microflora of aged FT as the focus of our study to further validate the role of important microorganisms. The bioaugmentation was a useful method to verify the function of microorganism without sterilizing raw materials. We picked the aged FT with quality defects as the subject of bioaugmentation test. The defective FT refers to FT that still have obvious quality defects after two years of spontaneous aging and cannot become qualified raw materials, which is a very common situation in the production process. In this study, the defective FCT was treated with bioaugmentation, which was different from the spontaneous aging process. Therefore, we used the paired comparison test to evaluate the effect of strains on the quality improvement of FCT. The control group was treated with equal amounts of sterile water under the same conditions. As shown in Fig. a, some quality traits were enhanced in the test samples by bio-inoculation with *Aspergillus nidulans* F4 for 48 h, with the total score improving by 2.5 points relative to the control group. 153 volatile compounds were detected by HP-SPME-GC/MS (Table A9). In Fig. 6b, the scatterplot of PCA (principal component analysis) of components demonstrated a significant difference between bio-inoculation and control group. All samples were in a 95% confidence interval. Thus, 29 DCs between the control and bioaugmentation group were identified. Figure 6c showed the differences in some flavor-related compounds, in which there were 8 terpenoids and degradation products, 4 alkane related to the higher fatty acid degradation and 4 aromatic compounds. Benzyl alcohol, benzaldehyde and phenethyl alcohol increased significantly. All terpenoids and degradation products were up-regulated except linalool. Neophytodiene and solanone are an important diterpenoid respectively derived from the degradation of chlorophyll and cembrene. The others are degraded from carotenoids, which could enhance the aroma intensity, pleasant odor and reduce irritation of FT [9]. They are flavor-related compounds and also found in good quality FT with spontaneous aging (shown in Fig. 2 and Table A1). The action of a microorganism on a compound is usually linked to the secretion of highly active enzymes. Figure 6d presents the results of a study of the enzymatic activity secreted by strain F4. We found that the enzyme activities of lipoxygenase (LOX) and peroxidase (POD) were higher than ones of other enzymes secreted by strain F4, which should be the reason why F4 can promote the degradation of fatty acids/esters and terpenoids, especially carotenoids.

## Discussion

By integrating the metabolome and microbiome, the role of microorganisms in quality improvement of FT through aging process has been investigated and is important for understanding the mechanism of the aging and reforming the traditional two-year aging process. With the non-targeted metabolomes based on three different platforms, volatile and non-volatile DCs were multi-dimensionally analyzed. The carbonyls were the most numerous and were positively correlated with quality. Most of the carbonyls were derived from terpenoids or aromatic compound metabolism. In terpenoid metabolism, polyterpenoids (e.g., limonene, zeatin, carotene, retinol and cembrene) degraded into megastigmatrienone, safranal, and dihydroactinidiolide and so on. Megastigmatrienone, named as "tabanone", was found as a key flavor compound in Burley tobacco, which gave FT a unique flavor and enhanced the smoke concentration [13]. Safranal could increase the sweetness and had been identified as an aroma compound by GC-O (gas chromatography–olfactometry) in Pu-erh tea [14]. Dihydroactinidiolide could mask bad flavors and enhanced cigarette palatability [15]. Previous studies have demonstrated that microorganisms could degrade terpenoids into aromatic components. The cocultivation of *Trichosporon asahii* and *Paenibacillus amylolyticus* were utilized to biodegrade lutein into the flavors existing in FT [16]. *Zygomycetes*, *Ascomycetes, Basidiomycetes* were also found to have the ability to degrade carotenoids [16, 17]. About the non-volatile and volatile acids, the reduction in higher fatty acid content (e.g., linolenic acid) in fatty acid metabolism could help to mellow the fragrance. Most esters/lactones also have a pleasant fruit or wine aroma to endow flavor in many foods [18]. Phenolic acid (e.g., chlorogenic acid, quinin acid, caffeic acid and so on) in amino acid or flavone metabolism, had been shown to play an important role in acid–base balance and improvement of smoke quality [19]. Proteins in tobacco could be degraded into amino acids, particularly aromatic amino acids, which were further converted into many aromatic compounds (e.g., benzene acetaldehyde, 2-methyl-benzaldehyde, 3-amino benzoic acid, and phenyl ethenone), and esterified to methyl phenyl acetate and methyl benzoate. The polysaccharides in tobacco (e.g., starch, cellulose, lignin, pectin and others) could be degraded into oligo- or mono-saccharides. Carbohydrates provided a carbon source and energy to keep microbes alive, and reduced irritation and mask undesirable odors. They might react with amino acids to form Maillard complexes with a nutty, sweet, and popcorn flavor [20].

Nicotine, as a major alkaloid in FT, was involved in nicotinate and nicotinamide metabolism. The right content of nicotine can enhance the flavor characteristics of cigarettes. However, the high nicotine content can cause irritating and even transform into tobacco-specific nitrosamines, which are harmful to human health [21]. In our study, the levels of nicotine, anatabinea, and nornicotine were all deceased, suggesting that the aging process contributed to reduce the harm of FT on humans. Several studies had also confirmed that the degradation of nicotine related to *Pseudomonas* sp., *Arthrobacter* sp., *Cellulomonas* sp., and also had the same metabolic intermediates (e.g., cotinine, nicotyrine, and nornicotine) found in our study [21].

Culture independent-based molecular methods provide the basis for probing the microbial diversity in FT. According to the alpha diversity indexes, the richness and diversity of bacteria and fungi in FT increased after the aging process, implicating that the microflora on FT remained active. In the bacterial flora, the composition of dominant genera differed from that of previous studies, which might be influenced by the different varieties or sources of FT [6, 7]. The dominant genera should be an important intervener of FT components. After aging, the relative abundance of *Pseudomonas* went from 2 to 18% with its strong tolerance to low moisture. *Pseudomonas* could degrade the nicotine of tobacco [22]. *Pseudomonas* and *Sphingomonas* could degrade aromatic amino acids [23]. *Sphingobacterium* along with *Acromobacter Xylosoxidans, Cupriavidus* were closely correlated with the formation of valine, glutamic acid and leucin [24, 25]. The fungal diversity of FT has been rarely reported. The dominant genera, *Aspergillus,* declined from 19 to 10% after aging, which might be affected by the consumption of oxygen in the aging stack. However, the role of *Aspergillus* in substrate degradation and flavor formation was unignored by secreting a variety of enzymes, similar to that of many traditional fermentation processes [26]. Additionally, the high abundance function categories of microbial flora were the amino acid metabolism, aromatic compound and secondary metabolite metabolism, which were in coincidence with DCs-related metabolic pathways. It suggested that the microorganisms with the ability to degrade terpenoids and higher fatty acid would help to promote the quality of FT.

According to the correlation analysis, *Acinetobacter* (B4), *Sphingomonas* (B15), and *Aspergillus* (F2) exhibited a closer relationship with the changes in DCs, were identified as the important genera mediated DCs according to the number of genera highly correlated with DCs. Among the interrelationships, *Acinetobacter* and *Sphingomonas* have exhibited an effective degradability of phenolic compounds or polyaromatic hydrocarbon [27]. While *Aspergillus* F2 was associated with the highest number of the degradation product of terpenoids and the higher fatty acids/esters. *Aspergillus* was also the dominant fungi in Pu-erh tea, and could change the composition of flavonoids and fatty acids [26]. In this study, the screened *Aspergillus nidulans* F4 was acted on the FT with quality defects by the bioaugmentation for 48 h. Most flavor precursors increased, which might be associated with the higher enzyme activities of LOX and POD secreted by F4. As in most of plants and fruits, norisoprenoids in FT were derived from an enzyme-catalyzed cleavage of the polyene chains of carotenes or xanthophylls, especially the carbon 13 compounds which act as potent flavor compounds [28]. Dioxygenases, such as LOX and POD, were thought to be one of the important members. LOX can biodegrade carotenoids with the co-oxidation by producing the hyperperoxides in intermediate states of peroxide [29]. Meanwhile, long-chain fatty acids can be converted into alkanes by LOX, which can relieve the irritation of FT raw materials. At the same time, norisoprenoid flavors from carotenoids also produced dihydroactinidiolide, safranal and β-cyclocitral by fungal POD [30]. Therefore, *Aspergillus nidulans* F4 could degrade terpenoids and higher fatty acids through the secretion of LOX and POD, thus contributing to the rapid improvement of FT quality. In contrast, the quality of study subject failed to meet the production requirements after two-year spontaneous aging, but now the quality score of samples with only 48 h of bioaugmentation could be increased by 2.5 points along with less quality defects, suggesting that *Aspergillus nidulans* F4 had an excellent ability to improve the quality of FT.

However, in our study, the diversity of tobacco-microbe was studied using next-generation sequencing, which only provided the analysis of the diversity of microbial community at the genus level. Therefore, it regrettably could not explain whether strain F4 was a major species of *Aspergillus sp.* at the molecular level. On the other hand, with the aid of the flow cytometric sorting techniques, we obtained the culturable filamentous strains at high efficiency form the aged FCT, in which strain F4 has a high frequency of emergence. Therefore, the bioaugmentation with the screened strain F4 could promote the FCT quality that explained, to some extent, the contribution of *Aspergillus sp.* to quality enhancement of FCT. It further also illustrated the important role of microorganisms in aging process for FT quality enhancement.

## Conclusion

By integrating the metabolome and microbiome, the role of microorganisms in quality improvement through FT aging process has been investigated. With the non-targeted metabolomes based on UPLC-QTOP-MS, GC–MS assisted derivatization, and HP-SPME-GC/MS platforms, 232 volatile and non-volatile DCs were comprehensively analyzed. The degradation products of terpenoids or higher fatty acids were one of the important factors in improving the FT quality. By microbiome, the diversity of tobacco-microbe was analyzed. The predicted high abundance function categories were in coincidence with DCs-related metabolic pathways. According to the correlation analysis, dominant genera and significant DCs exhibited a close relationship. At last, the 48 h of bioaugmentation with the screened *Aspergillus nidulans* F4 verified the role of a microorganism in the quality enhancement of FCT. Our findings could provide a useful reference for the profiling the microbial roles in the spontaneous process and provide an important information on choosing functional strains for the aging process of FT or other plant material.

## Methods

### Sampling

UAFT and AFT were samples collected at the beginning and at two years of the spontaneous aging, separately. Samples were collected using a five-point method from six different containers, at China Tobacco Si Chuan industrial Co, Ltd (Chendu County, Sichuan Province, China), as shown in Fig. A5. Finally, the samples were frozen immediately in liquid nitrogen and stored at -80 °C.

### Profiling DCs between UAFT and AFT

The non-volatile and volatile components of FT were comprehensively determined with the non-targeted metabolomics based on UPLC-QTOP-MS, GC–MS assisted derivatization and HP-SPME-GC/MS platform. Before measurement, samples were ground for 90 s under 60 Hz by TL- 48R grinder (TL-48R, Jingxin, ShangHai, China).

#### UPLC-QTOP-MS analysis

A 0.05 ± 0.01 mg of the samples powder was extracted with 1 mL of single-phase solvent (acetonitrile-methanol–water, 2:2:1, 1 μg·mL^−1^ 2-Chloros-L-phenylalanine as an internal standard, ≥ 98%, Sigma-Aldrich). The samples were vortexed for 30 s, sonicated for 5 min in an ice-water bath, incubated at -20 °C for 1 h, and centrifuged at 4 °C and 10,000 × g for 15 min. The resulting supernatants were gathered into vials for analysis.

Non-volatile components were analyzed with an UHPLC system (1290-Agilent Technologies) coupled with a UPLC HSS T3 column (2.1 mm × 100 mm, 1.8 um, Waters) and Q Exactive mass spectrometer (QE MS, Orbitrap MS, Thermo Fisher Scientific). The gradient elution was performed using a mobile phase A and phase B (acetonitrile, LC–MS grade, CNW Technologies). The phase A was respectively 0.1% v/v formic acid solution and 5 mmol·L^−1^ ammonium acetate solution in positive (POS) and negative (NEG) ion mode (LC–MS grade, CNW Technologies). With 2 μL injection volume, the flow rate of mobile phase was 0.5 mL·min^−1^, and the elution gradient was programmed as follows:1% B (v/v) from 0 to 1 min, 1%-99% B (v/v) from 1.1 to 10 min, 99%-1% B (v/v) from 10.1 to 12 min [12]. The QE mass spectrometer was used to gain MS/MS spectra based on a simultaneous scan POS and NEG and information dependent acquisition (IDA) triggered the enhanced ion scan (EPI) mode. The conditions of the electro spray ionization source were that the sheath and aux gas flow rate were 45 Arb and 15 Arb, respectively, the capillary temperature was 400 °C, the full MS resolution was 70,000, the MS/MS resolution was 17,500, the collision energy was 20/40/60 eV, and the spray voltage was 4.0 kV in POS or -3.6 kV in NCE. The MS data was evaluated continuously by the acquisition software (Xcalibur 4.0.27, Thermo). The raw data were transformed into mzXML format by ProteoWizard and processed by MAPS software (version 1.0). The pre-processing results generated a data matrix comprising the peak intensity, retention time (RT), and massto-charge ratio (m/z) values. Finally, the compounds were identified by the in-house MS2 database (Shanghai Biotree biotech CO., Ltd., Shanghai, China).

#### GC–MS assisted derivatization analysis

A 20 ± 1 mg of powder was extracted into 1 mL of solvent (acetonitrile-methanol–water, 2:2:1, 1 μg·mL^−1^ adonitol as the internal standard, LC–MS grade, ≥ 99%, SIGMA). Samples were treated in the same way as the above method. The 0.2 mL resulting supernatant was dried in a vacuum concentrator (LNG-T98, Huamei, Taichang, China). The residue was added into 80 μL methoxyamination hydrochloride solution (20 g·L^−1^, dissolved in pyridine, Supelco, Aladdin), incubated at 80 °C for 30 min and derivatized with 100 μL of 1% trifluoroacetamide (≥ 98.0%, Supelco, Aladdin) at 70 °C for 1.5 h.

After cooling, the samples were analyzed with an Agilent 7890-Pegasus HT GC/MS system equipped with an Agilent G4513A automatic injector (Agilent, USA, LECO, USA) and Agilent DB-5MS column (30 m × 250 μm × 0.25 μm). 1 μL aliquot of sample was injected using the splitless mode. The temperatures of injection, transfer line, and ion source were 280 °C, 280 °C, and 250 °C, respectively. The temperature gradient of the oven was that the initial temperature was at 50 °C for 1 min, then rase to 310 °C at a rate of 10 °C per min and maintained for 8 min. The carrier gas was helium, the front inlet purge flow was 3 mL·min^−1^, and the gas flow rate through the column was 1 mL·min^−1^. The ionization mode was the electron impact, and the energy was -70 eV. The mass spectrometry data were obtained in full-scan mode with full scanning range of 50–500 min, a rate of 12.5 spectr·s^−1^ and a solvent delay 6.25 min. Raw data were processed using ChromaTOF software (V4.3xLeco) including peak extraction, baseline adjustment, deconvolution, alignment and integration. The compounds were identified by matching the mass spectrum and retention index in LECO-Fiehn Rtx5 database and the in-house MS2 database (Shanghai Biotree biotech CO., Ltd., Shanghai, China) [31].

#### HP-SPME-GC/MS analysis

A 2.00 ± 0.01 g of FT powder with 1 μL tritiated naphthalene (2 mg·mL^−1^, dissolved in dichloromethane, Supelco, Aladdin) as internal standard was transferred into the 20 mL headspace bottle. Volatiles in FT were extracted for 30 min at 60 °C by SPME fiber assembled with DVB/CAR/PDC (divinylbenzene/carboxen/polydimethylsiloxane, 50/30 μm, Supelco, ANPEL Laboratory Technologies (Shanghai) Inc.). The equipment was the same as that described above in GC–MS assisted derivatization analysis. The chromatographic conditions were as follows: column flow of 1 mL·min^−1^, injection temperature of 250 °C, holding at 40 °C for 2 min, heating up to 250 °C at the rate of 10 °C·min^−1^ and holding for 6 min, ion source adopting an electron bombardment model with an electron energy of 70 eV, delivery line temperature of 280 °C and ion source temperature of 210 °C, mass spectrometry data in full scan mode with 33–400 atomic mass units in full scan mode with a data acquisition rate of 10 specs·s^−1^. The compounds were identified by the mass spectrometry data blasting into the MS library of the Wiley library (NY, version 9.0) and National Institute for Standards and Technology (NIST, Search Version 1.6). The identified compounds with the matching scores above 700 were kept for further analysis. Additionally, the compounds were identified again by the retention index. The mixture of C5-C30 n-alkanes was as the mixed standard and analyzed under the same conditions. According to the retention time of each alkane, the retention index of each compound was calculated with the improved Kovats' method [32], then compared with the retention index in the database. All samples were conducted in sextuplicate. At last, the peaks detected in more than half of samples or in samples with RSD (relative standard deviation) < 30% were retained [31].

#### Identification of DCs and mapping of relevant metabolic pathways

Via SIMCA software (v 14.1, MKS Umetrics AB), an OPLS-DA model was constructed with the quality evaluation score (as Y-axis) and the relative content of the compositions (as X-axis). The DCs between the UAFT and AFT samples were identified depending on the VIP value > 1 and *p* value < 0.05. The selected DCs were mapped on the metabolic pathways in the KEGG libraries to identify the enriched pathway.

### Evaluation of quality

We organized a panel of seven professional assessors, including two females and five males, to evaluate the quality of the samples in strict accordance with the recommended standards of Chinese tobacco industry (YC/T138-1998, YC/T496-2014). There are eight evaluation indicators, including aromatic quality, aromatic intensities, pleasant odor, smoke strength, smoke intensities, softness, aftertaste, and sweetness. The total score of quality evaluation is the sum of the scores of each indicator.

### Profiling of microbial diversity

#### DNA extraction and PCR amplification

A 10 ± 0.1 g of each sample mixed with 200 mL sterilized phosphate buffer solution (PBS, 0.1 mol·L^−1^, pH 7.2) and shocked at 220 rpm, 30 °C for 2 h, sonicated for 5 min and filtered with the sterile absorbent gauze. The result filtrate was centrifuged to collect sediment at 10,000 × g for 10 min. At last, the metagenomic DNA of microflora in FT was extracted with the DNeasy PowerSoil Kit (QIAGEN, Inc., Netherlands) according to the instructions.

After quantitatively and qualitatively analyzed by 1.2% agarose gel electrophoresis, DNA extraction was used as the template for amplification. V4-V5 hypervariable region of the bacteria 16S rRNA gene was amplified with the barcoded universal primers (515F: 5'- GTGCCAGCMGC CGCGGTAA-3', 907R: 5'-CCGTCAATTCMTTTRAGTTT-3'). In addition, the intergenic transcribed spacers of the fungal rRNA gene were amplified with the primer (ITS1F: 5'- CTTG GTCATTTAGAGGAAGTAA-3', ITS2R: 5'-GCTGCGTTCTTCATCGATGC-3'). The polymerase chain reactions were amplificated in 30 µL reactions with 15 µL of Phusion High-Fidelity DNA polymerase (TransGen Biotech, China), 10 ng of DNA template and 0.2 µM of forward and reverse primers [12]. The programs of PCR (Polymerase Chain Reaction) were initial denaturation at 98 °C for 2 min, 30 cycles of denaturation at 98 °C for 15 s, annealing at 55 °C for 30 s, elongation at 72 °C for 30 s, finally extension at 72 °C for 5 min. Subsequently, the amplified products were purified, recovered using magnetic beads (Vazyme VAHTSTM DNA Clean Beads) and quantified by fluorescence with a microplate reader (BioTek, FLx800, USA).

#### Microbial diversity profiling

An equal amount of the amplicons was sequenced using paired-end 2 × 250 bp on the Illlumina MiSeq platform with MiSeq Reagent Kit V3 (Illumine, USA). The bioinformatics of raw sequence was analyzed with QIIME2 [33]. Simply, the raw sequence data was processed by DADA2 method [34] including depriming, quality filtering, denoising, splicing and chimera removal. Through quality control, each deduplicated sequence with a cluster similarity close to 100% was termed ASV [35]. The taxonomy of ASVs was obtained by blasting against the Silva database (Release 13.8,) for the bacterial 16S rRNA genes and against the UNITE database (Release 8.0, https://unite.ut.ee/) for the fungal ITS1 genes. With the rarefaction method, the sequencing data of each sample was flattened by randomly extracting sequences from each sample to reach a uniform depth for validly predicting the ASVs and their relative abundances. The depth level was set at the 95 percent of the minimum amount of sample sequence.

#### Microflora function prediction

Based on the sequence of 16S rRNA and ITS, the microflora functions were predicted by PICRUSt2 software package (https://github.com/picrust/picrust2/wiki). Compared to the initial version, PICRUSt2 was improved with a 10 times larger reference genome database and added MetaCyc metabolic pathway (https://metacyc.org/). MetaCyc database is widely used to predict both primary and secondary metabolic pathways as a nonredundant, intensively curated, and comprehensive database, which can be near to the results of macrogenomic data [36].

### Correlation analysis between dominant genus and DCs

Using the OPLS-DA model, the top 30 bacterial and top 20 fungal genera based on the relative abundance were selected for the analysis of differential genera Then, the interrelation between differential genera and DCs was further explored using a Spearman's correlation analysis. The well-correlated members (|*r*|> 0.7 and *p* < 0.05) were visualized as the positive and negative relationship of associated networks with edge-weighted layouts by Cytoscape (v 3.7.1) software.

### Validation of correlation relationships

Refer to the previous sorting methods [37], we first sorted single cells of tobacco-microbe from a multi-strain suspension into 96-well plates using flow cytometry (FACSAria III Cell Sorter, BD Biosciences, USA). Briefly, 10 ± 0.1 g of FT sample was added into 200 mL of sterilised phosphate buffer solution (PBS, 0.1 mol/l pH 7.2), shaken and filtered. The result filtrate was centrifuged at 7,000 × g for 10 min. The collected multi-strain suspension was resuspended in the sterile PBS, then filtered by a 40 μm filter and diluted to the required absorbance (OD_600_ = 0.3). At last, 1 mL of the multi-strain suspension was transferred into a sample cell of flow cytometry. By isolating the mixed strains, single-cells were transferred to 96-well plates filled with Bengal Red (BR) Agar and incubated at 30 °C for 72 h. Filamentous strains were identified by colony morphology and then transferred to 96 deep well plates filled with BR broth and cultured with shaking at 400 rpm for 72–120 h. The BR broth, added with 20% (v/v) FT extract, was a yellowish-brown color which has a strong absorbance at 460 nm by spectrophotometry. Based on the colony and cell morphology of the strains, some strains with mycelium could be presumed to be *Aspergillus spp*. Strains that could lighten the color of the medium or reduce absorbance were selected out as further research objects, among which the strain named F4 has a high frequency of emergence and the ability to lighten the color of FT extracts.

Colony PCR of strain F4 was performed to amplify the ITS1-5.8S rRNA-ITS2 region with the primers ITS1 (5′-TCCGTAGGTGAACC TGCGG-3′) and ITS4 (5′-TCCTCCGCTTATTGATATGC-3′). The qualified PCR products were sent to Sangon Biotech Co., Ltd. (Shanghai, China) for sequencing. With the help of the Basic Local Alignment and Search Tool (BLAST) algorithm, the sequence from the isolated strains F4 were compared with sequences in NCBI GenBank database (National Center for Biotechnology Information, https://blast.ncbi.nlm.nih.gov/) to identify. The sequence ident between strain F4 and *Aspergillus nidulans* ATCC 10,074 was 100%.

Then, strain F4 was multiplied in Red Bengal liquid medium with 20% (v/v) FT extract at 30 °C, 220 rpm·min^−1^ for 72 h. To investigate the role of strain F4, a comparative study was performed with test and control groups. The test group was that the resulting suspension of strain F4 was sprayed onto the surface of FT at 20% (v/w) and cultured at 30 °C, 85% humidity and stirred per four hours. Under the same conditions as the test group, the control group was FT processed with the sterile distilled water. The defective FT, with strong irritation and insufficient aroma, has been treated with the spontaneous aging in the factory for two years, but the quality failed to meet the production requirements, which is commonly found in industrial. At last, FT samples were frozen in liquid nitrogen, collected into sterile polyethylene bags and stored at -80 °C.

In order to further study the function, the enzymatic activity secreted by F4 was measured. The activity of LOX (EC1.13.1.13) was determined using the linoleic acid assay [38]. One unit of LOX activity (U) was defined as an increase of 0.1 of absorbance at 234 nm per minute. The activity of POD (EC 1.11.1.7) was determined by the guaiacol assay [39]. One unit of POD activity (U) was defined as an increase of 0.01 unit of absorbance at 470 nm per minute. The activity of polyphenol oxidase (PPO, EC 1.10.3.1) was measured with the catechol assay [40]. One unit of PPO activity (U) was defined as an increase of 0.01 unit of absorbance at 420 nm per minute. Results were expressed as units per mL of fermented supernatant by strain F4 (U·mL^−1^).

### Statistical analysis

All samples conducted at least in triplicate and data presented as mean ± standard error of the mean (SEM). R software (v 3.6.2) was used to calculate the Spearman's correlation coefficient, *p*-values, and drew the hot map.

## Supplementary Information

**Additional file 1:** **Fig. A1.** Total ion chromatograms of UAFT and AFT. **Fig. A2.** OPLS-DA analysis for UAFT and AFT samples. **Fig. A3.** Differential analysis of components between UAFT and AFT by OPLS-DA model. **Fig. A4.** Cross-validation plot of OPLS-DA model for different genus between UAFT and AFT. **Fig. A5.** Protocol of sample collection.

**Additional file 2:** **Table A1.** Summary of differential compounds of UAFT and AFT. **Table A2.** V3-V4 16S rRNA sequence reads of microbiome in UAFT and AFT. **Table A3.** ITS1 sequence reads of microbiome in UAFT and AFT. **Table A4.** Alpha diversity of bacterial flora. **Table A5.** Alpha diversity of fungal flora. **Table A6.** Composition of bacterial metabolic pathways based on the first-level and second-level functions in the MetaCyc. **Table A7.** Composition of fungal metabolic pathways based on the first-level and second-level functions in the MetaCyc. **Table A8.** the taxonomic compositions and difference analysis of microbial community. **Table A9.** Summary of differential compounds of bioaugmentation with F4 and control group.

## Data Availability

All data generated or analyzed during this study are included in this published article and its additional files. The raw reads of FT samples were submitted to NCBI Sequence Read Archive (SRA) database (SRA, https://www.ncbi.nlm.nih.gov/sra) (accession number PRJNA638231). The nucleotide sequence of strain F4 was submitted to GenBank (accession number MZ452629).

## Abbreviations

FT: Flue-cured tobacco
DCs: Differential compounds
AFT: The aged flue-cured tobacco
UAFT: The unaged flue-cured tobacco
UPLC-QTOP-MS: The ultra-performance liquid chromatography quadrupole time-of-flight mass spectrometry;
GC–MS assisted derivatization: Gas chromatography-mass spectrometer assisted derivatization
HP-SPME-GC/MS: Headspace solid-phase micro-extraction assisted GC–MS
VIP: Variable importance for predictive components
POS: Positive ion pattern
NCE: Negative ion pattern
LOX: Lipoxygenase
POD: Peroxidase
CPM: Read counts per million
ASVs: Amplicon sequence variants
OTUs: Operational taxonomic units
OPLS-DA: Orthogonal Partial Least Squares Discriminant Analysis
PICRUSt2: Phylogenetic Investigation of Communities by Reconstruction of Unobserved States
KEGG: Kyoto Encyclopedia of Genes and Genomes
CN: The control group
