# Supplementary material for: Profiling the role of microorganisms in quality improvement of the aged flue-cured tobacco
Source: BMC Microbiol. 2022 Aug 15;22:197. doi: 10.1186/s12866-022-02597-9 (PMC9377114; doi:10.1186/s12866-022-02597-9)
Supplement: Supplementary file 1 — Additional file 1: Fig. A1. Total ion chromatograms of UAFT and AFT. Fig. A2. OPLS-DA analysis for UAFT and AFT samples. Fig. A3. Differential analysis of components between UAFT and AFT by OPLS-DA model. Fig. A4. Cross-validation plot of OPLS-DA model for different genus between UAFT and AFT. Fig. A5. Protocol of sample collection. [file 12866_2022_2597_MOESM1_ESM.pdf]

Profiling the role of microorganisms in quality improvement of the aged flue-cured tobacco

Xinying Wu[1,2,3], Wen Cai[4], Pengcheng zhu[1,4], Zheng Peng[1,2], Tianfei Zheng [1,2], Dongliang Li[4] Jianghua Li[1,2],

Guanyu Zhou [1,2], Guocheng Du[1,5*], Juan Zhang[1,2 *]

[1] School of Biotechnology, Jiangnan University, 1800 Lihu Road, Wuxi 214122, China

[2]Science Center for Future Foods, Jiangnan University,1800 Lihu Road, Wuxi, 214122, China

[3]School of Liquor and Food Engineering, Guizhou University, Guiyang 550025, China

[4] Technical Research Center, China Tobacco Sichuan industrial Co., Ltd.,56 Chenglong Road, Chengdu 610000, China

[5] The Key Laboratory of Carbohydrate Chemistry and Biotechnology, Ministry of Education, Jiangnan University,1800 Lihu Road, Wuxi, 214122, China

12

***Correspondence:**

gcdu@jiangnan.edu.cn (D.G.C.), Tel.: +86-510-85918309

zhangj@jiangnan.edu.cn (Z.J.), Tel.: +86-510-85918309

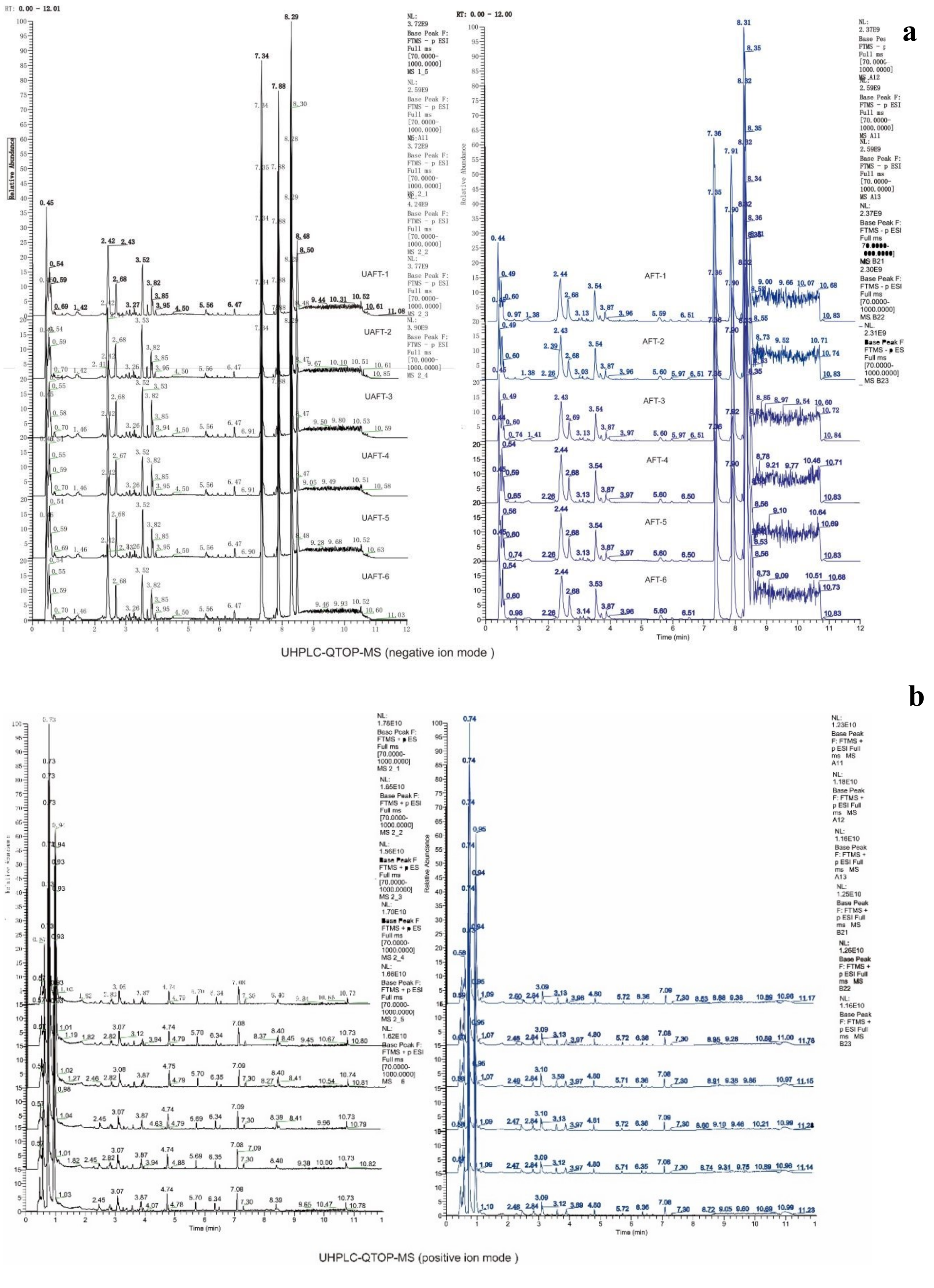
a
UAFT-1
UAFT-2
UAFT-3
UAFT-4
UAFT-5
UAFT-6
AFT-1
AFT-2
AFT-3
AFT-4
AFT-5
AFT-6
Relative Abundance
Time (min)
UHPLC-QTOP-MS (negative ion mode)
b
Relative Abundance
Time (min)
UHPLC-QTOP-MS (positive ion mode)

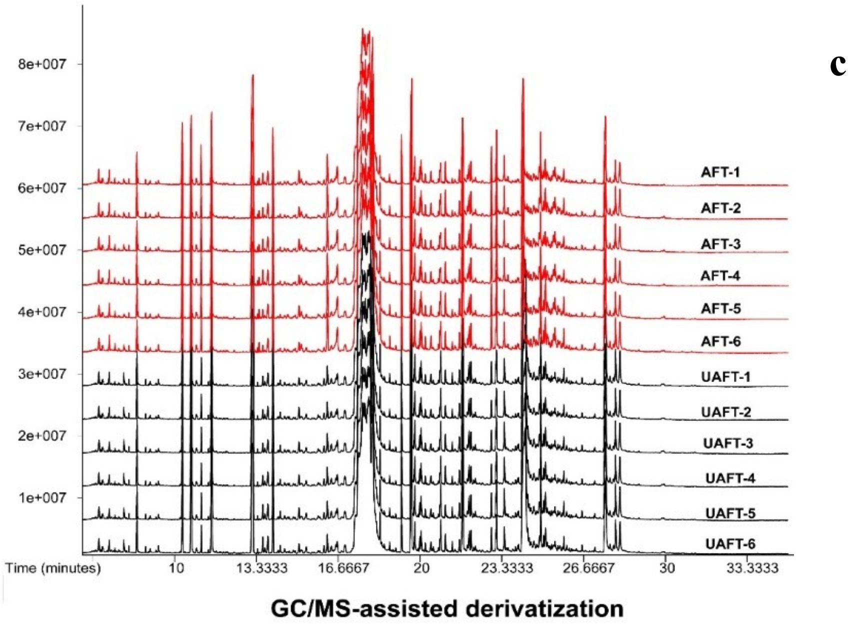

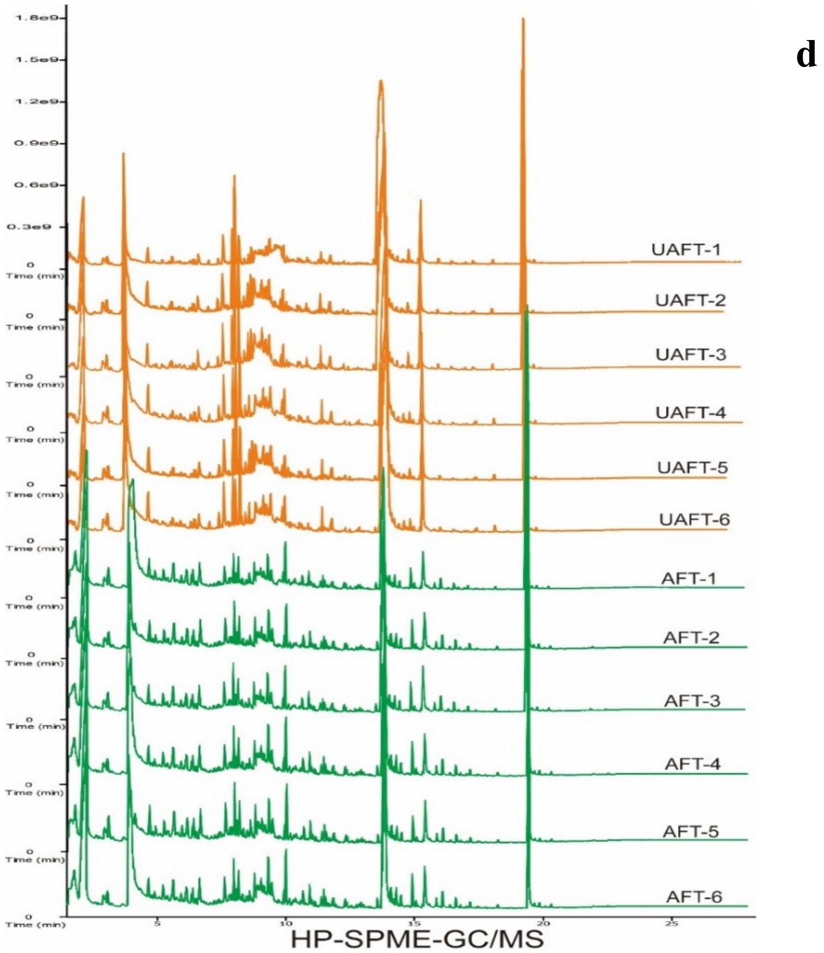

Fig. A1 Total ion chromatograms of UAFT and AFT

a: Total ion chromatograms of HP-SPME-GC/MS; **b**: Total ion chromatograms of GC/MS-assisted derivatization; **c**: Total ion chromatograms of UHPLC-QTOP-MS in negative; **d**: Total ion chromatograms of UHPLC-QTOP-MS in positive ion mode (d)

AFT represents the aged flue-cured tobacco. UAFT represents the unaged flue-cured tobacco.

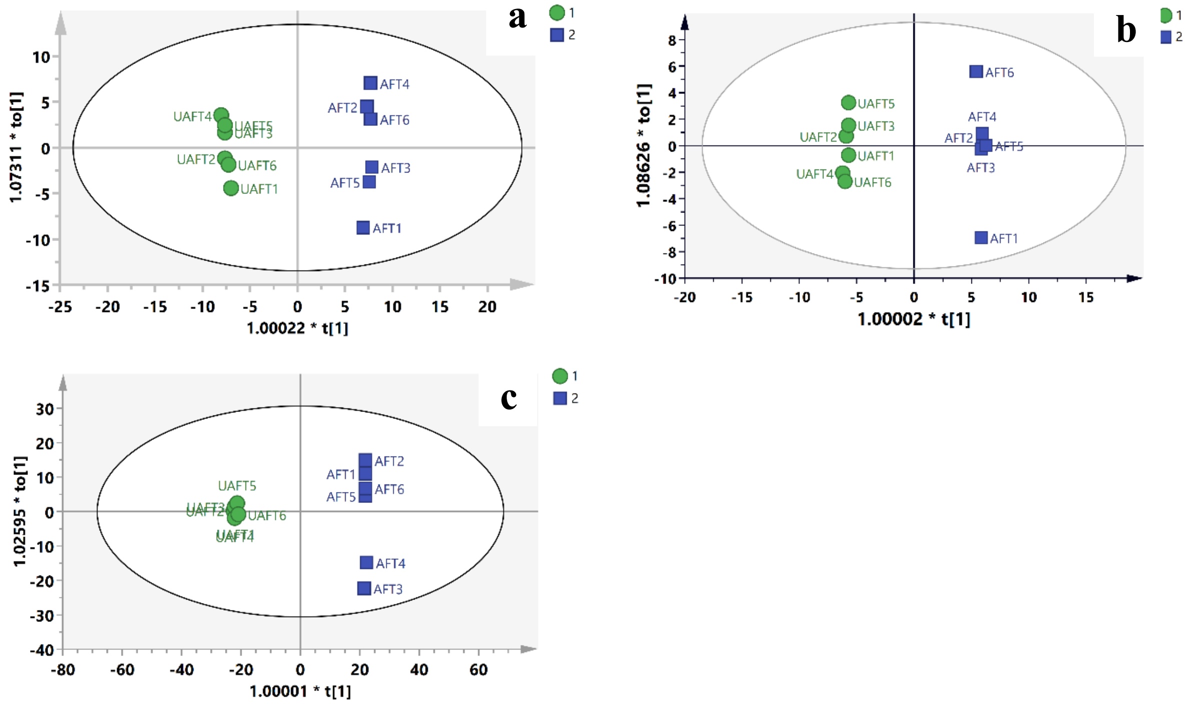

Fig. A2 OPLS-DA analysis for UAFT and AFT samples

a: OPLS-DA analysis based on UPLC-QTOP-MS; **b**: OPLS-DA analysis based on GC-MS assisted derivatization **c**: OPLS-DA analysis based on HP-SPME-GC/MS

AFT represents the aged flue-cured tobacco. UAFT represents the unaged flue-cured tobacco.

UAFT1-6 represents six duplicate samples of UAFT; AFT1-6 represents the six duplicate samples of AFT.

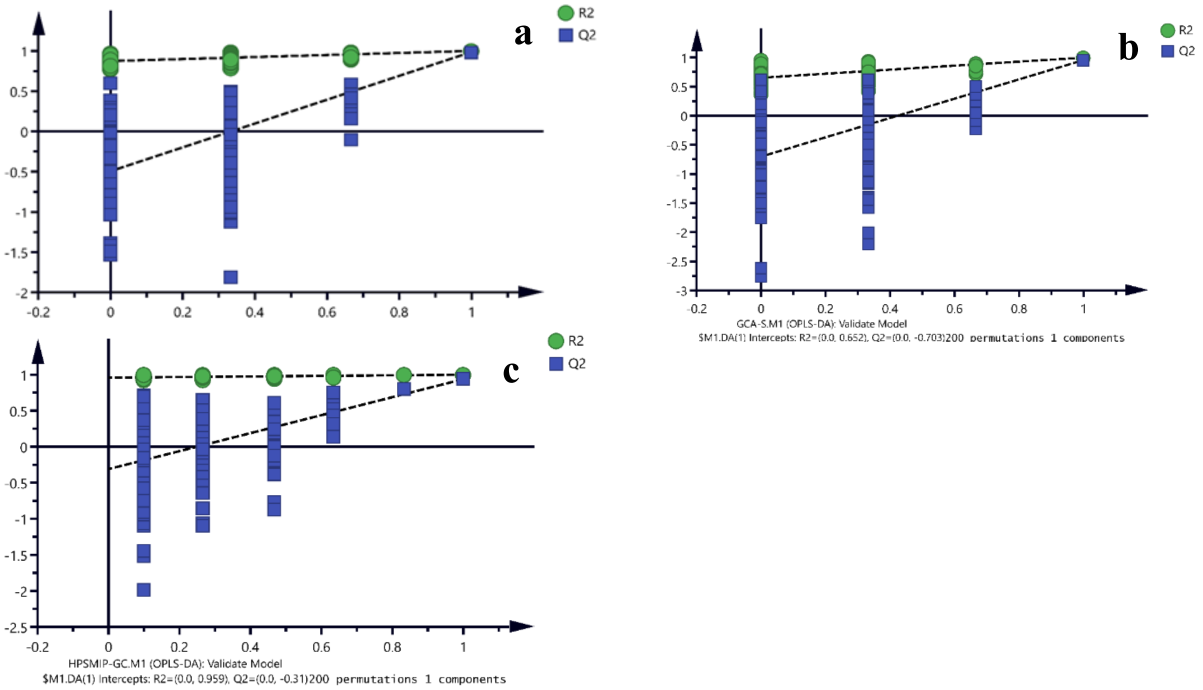

Fig. A3 Differential analysis of components between UAFT and AFT by OPLS-DA model,

a: the cross-validation plot of OPLS-DA model with 200-time permutation tests based on UPLC-QTOP-MS (the intercepts of R2 and Q2 were 0.874 and -0.494 respectively); **b**: permutation tests based on GC-MS assisted derivatization (the intercepts of R2 and Q2 were 0.652 and -0.703, respectively); **c**: permutation tests based on HP-SPME-GC/MS (the intercepts of R2 and Q2 were 0.959 and -0.31, respectively)

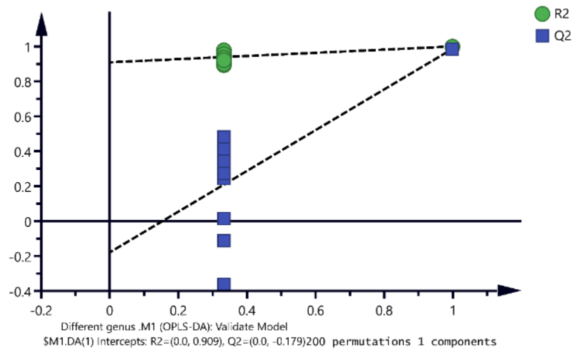

Fig. A4 Cross-validation plot of OPLS-DA model for different genus between UAFT and AFT

OPLS-DA model with 200-time permutation tests (the intercepts of R2 and Q2 were 0.909 and -0.179 respectively)

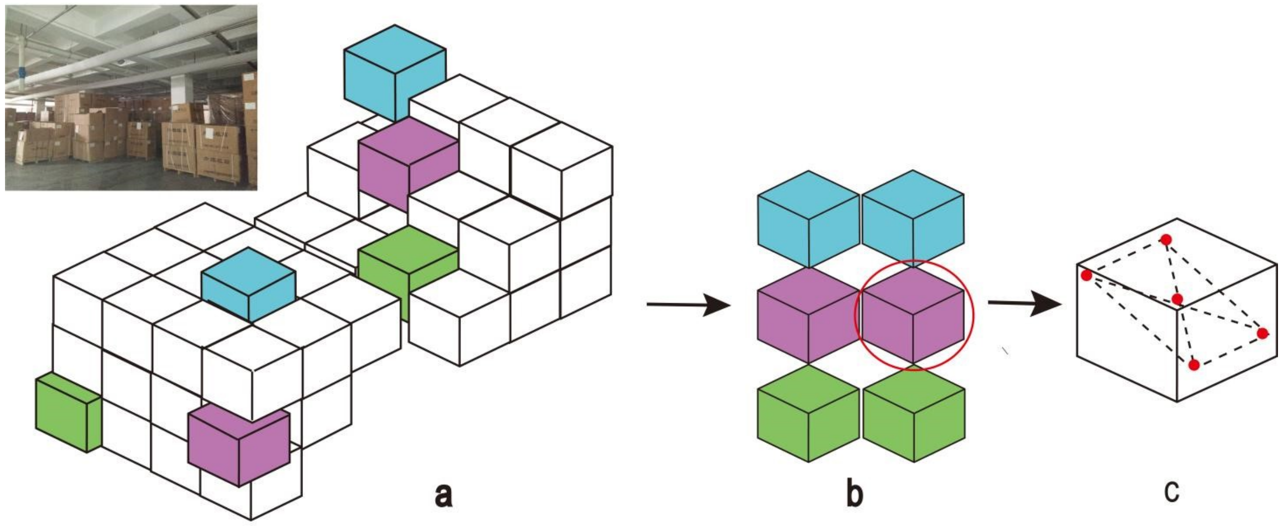

Fig. A5 Protocol of sample collection

a: selecting the fixated stockpile of FCT in the aging storage; **b:** selecting six different containers from the top, middle and bottom position of stockpile; **c:** sampling from the center and four corner positions of every aging box
